# Toxicity, Spectroscopic Characterization and Electrochemical Behaviour of New Macrocclic Complexes of Lead(II) and Palladium(II) Metals

**DOI:** 10.1155/MBD.2000.211

**Published:** 2000

**Authors:** Anil Bansal, Randhir Singh, R. V. Singh

**Affiliations:** 1 Department of Chemistry University of Rajasthan Jaipur 302004 India; 2 Department of Chemistry Gurukula Kangari Vishwavidyalaya Hardwar 249404 (U.P.) India

## Abstract

Tetraazamacrocyclie complexes of lead and palladium have been synthesized by the template process using the bis(benzil)ethylenediamine precursor. The tetradentate macrocycle (maL) reacts with PbCl_2_, PdCl_2_ and different diamines in a 1:1:1 molar ratio in methanol to give several solid complexes of the types [Pb(maL)(R)Cl_2_] and [Pd(maL)(R)]Cl_2_ (where R = 2,6-diaminopyridine or 1,2-phenylenediamine). The macrocycle and its metal complexes have been characterized by elemental analysis, molecular weight determinations, molar conductivity, IR, ^1^H NMR, ^13^C NMR, electronic, mass and electrochemical studies. The macrocyclic ligand coordinates through the four azomethine nitrogen atoms which are bridged by benzil moieties. IR spectra suggest that the pyridine nitrogen is not coordinating. The palladium complexes exhibit tetracoordinated square-planar geometry, whereas a hexacoordinated octahedral geometry is suggested for lead complexes. The macrocycle along with its complexes have been screened *in vitro* against a number of pathogenic fungi and bacteria to assess their growth inhibiting potential.


### TOXICITY, SPECTROSCOPIC CHARACTERIZATION AND ELECTROCHEMICAL BEHAVIOUR OF NEW MACROCYCLIC COMPLEXES OF LEAD(II) AND PALLADIUM(II) METALS.

Anil Bansal[1], Randhir Singh[2] and R.V. Singh*[1]

[1]Department of Chemistry, University of Rajasthan, Jaipur – 302004, India
[2] Department of Chemistry, Gurukula Kangari Vishwavidyalaya, Hardwar – 249404 (U.P.), India

### ABSTRACT

Tetraazamacrocyclic complexes of lead and palladium have been synthesized by the template process using the bis(benzil)ethylenediamine precursor. The tetradentate macrocycle (maL) reacts with $PbCl_2$, $PdCl_2$ and different diamines in a 1:1:1 molar ratio in methanol to give several solid complexes of the types [Pb(maL)(R)$Cl_2$] and [Pd(maL)(R)]$Cl_2$ (where R = 2,6-diaminopyridine or 1,2-phenylenediamine). The macrocycle and its metal complexes have been characterized by elemental analysis, molecular weight determinations, molar conductivity, IR, $^1$H NMR, $^{13}$C NMR, electronic, mass and electrochemical studies. The macrocyclic ligand coordinates through the four azomethine nitrogen atoms which are bridged by benzil moieties. IR spectra suggest that the pyridine nitrogen is not coordinating. The palladium complexes exhibit tetracoordinated square-planar geometry, whereas a hexacoordinated octahedral geometry is suggested for lead complexes. The macrocycle along with its complexes have been screened *in vitro* against a number of pathogenic fungi and bacteria to assess their growth inhibiting potential.

### INTRODUCTION

The field of macrocyclic chemistry of metals is developing very fast because of its variety of applications[1] and importance in the area of coordination chemistry[2]. The rational design and construction of inorganic and organometallic metallomacrocycles by transition metal-directed multicomponent self-assembly has a major impact on supramolecular chemistry[3,4]. The incorporation of metal centres into supramolecular systems gives rise to novel electronic and/or magnetic properties as well as fascinating structural features. Metallomacrocycles have been widely used in host-guest chemistry to understand self-assembly phenomena in natural systems[4,5]. Transition metals present a large variety of preferred coordination geometries which have been systematically used in the construction of supramolecular assemblies such as molecular racks[6], ladders[6], girds[7], cyclinders[8], metallomacrocycles[5,9], metallocyclic polygons[10] and helicates[11].

The importance of macrocyclic complexes is now well recognised. In the past, attention has been paid on the design and synthesis of small molecular complexes that mimic aspects of the spectral and chemical properties of metal sites proteins. The desire to find highly water soluble and stable redox couple based on inexpensive materials for use as components in photoelectrochemical cells and redox storage batteries had led us to investigate bipyridine complexes of palladium. One of the most interesting aspects of polyazamacrocyclic complexes is that the ligand can be modified. Some transition metal(II) complexes of polyazamacrocyclic ligands containing coordinated secondary amino groups are chemically oxidized to metal(II) compelxes containing a higher degree of unsaturation in the ligand[12,13]. The reaction is largely affected by the nature of the central metal ion and the structure of the ligand. The effect of macrocyclic and chelate-ring size on the complexation behaviour of a series of tetraazamacrocycles has been investigated[14]. Change in ring size has been achieved solely by varying the number of methylene carbons that link the nitrogens atoms to the benzyl moiety. The coordination chemistry of square-planar metal complexes involving nitrogen donor ligands has excited great interest among chemists in recent years, due to the applications of these compounds in catalysis[15] and their relevance to bioinorganic systems. For instance, the complexes of platinum(II) and palladium(II) exhibit potent antitumor activity[16]. It is known that activity is associated with the configuration of the platinum(II) and palladium(II) complexes[17]. Macrocyclic ligands are chemically interesting as they show metal exchange (transmetallation) reactions which are useful for the synthesis of new metal complexes. A current review[18] on synthetic chlorophyll and haemoglobin reveals the importance of polyazamacrocycles as oxygen carriers. Transition metals and their complexes have great interest and form mutliple complexes[19]. Transition metal complexes of nitrogen donor ligands have been studied in detail, on account of their stereochemistry and wide practical utility[20].

The striking structural features, diverse stereo-chemistry and appreciable biochemical as well as commercial applications of palladium(II) and lead(II) complexes of biologically active nitrogen donor ligands led us to undertake systematic studies on the complexes of these two metals. The synthesis of some new complexes of palladium(II) and lead(II) with the macrocycle and their structure elucidation and redox behaviour are reported in this communication.

### MATERIAL AND METHODS

The chemicals used were of AR grade. These chemicals and solvents were dried and purified by standard methods.

**Preparation of Bis(benzil)ethylenediamine**

Bis(benzil)ethylenediamine was prepared from ethanolic solutions (35ml) of benzil(5.3508 g, 25.4 mmol) and 25 ml solution of ethylenediamine (0.7648 g, 12.7 mmol) in ethanol. The reaction mixture was refluxed for 3h. After reducing the solvent, the soltuion was cooled and the reddish yellow crystalline compound thus obtained was recrystallized from ethanol.

[$C_{30}H_{24}N_2O_2$], Found % : C, 80.76; H, 5.32; N, 5.92, Calcd. % : C, 81.15; H, 5.44; N, 6.30; mol. wt., Found. 412, Calcd. 444; m.p., 86-89°C; $^1H$ NMR (DMSO-$d_6$): δ3.12 (bs,-$NCH_2$) and aromatic protons δ8.00 (d,2a), δ7.36 (dd, 3a) and δ7.65 (d, 4a).

**Preparation of the Macrocyclic Complexes**

Bis(benzil)ethylenediamine (0.6983g, 1.57 mmol) and 2,6-diaminopyridine (0.1716g, 1.57 mmol) were mixed in a minimum quantity of MeOH and a MeOH solution of the metal salt (0.4369 g, 1.57 mmol) was added. The reaction mixture was heated under reflux for 6h. The reaction solution was concentrated to half volume. After cooling, the solution was transferred to an evaporating dish and kept overnight at room temperature. The blackish crystals separated out were collected, washed with hot water, then with cold methanol, dried under reduced pressure and recystallized from methanol / benzene 1/1.

**[Pb($C_{35}H_{27}N_5Cl_2$]** : Found % : C, 52.42; H, 3.28; N, 8.43; Cl, 9.29; Pb, 25.59, Calcd. % : C, 52.81; H, 3.42; N, 8.80; Cl, 8.91; Pb, 26.03; mol. wt. Found : 775; Calcd. : 796; m.p., 144°C; $^1H$ NMR (DMSO-$d_6$) : δ3.33 (bs, -$NCH_2$), δ7.52-7.63 (R=Py.$C_5H_7N_3$), and aromatic protons : δ7.63 (d, 2a), δ7.52 (dd, 3a), δ7.58 (d, 4a).

Similarly, by using $PdCl_2$ and the respective amines, the complexes [Pb($C_{36}H_{28}N_4$)$Cl_2$], [Pd($C_{35}H_{27}N_5$)]$Cl_2$ and [Pd($C_{36}H_{28}N_4$)]$Cl_2$ were also prepared.

**[Pb($C_{36}H_{28}N_4$)$Cl_2$]** : Found % : C, 53.95; H, 3.65; N, 7.39; Cl, 8.50; Pb, 25.65, Calcd. % : C, 54.39; H, 3.55; N, 7.05; Cl, 8.92; Pb, 26.06; mol. wt. Found : 814; Calcd. : 795; m.p., 130°C; $^1H$ NMR (DMSO-$d_6$) : δ3.29 (bs,-$NCH_2$), δ7.24-7.35 (R=Ph.$C_6H_8N_2$), and aromatic protons : δ7.35 (d, 2a), δ7.24 (dd, 3a), δ7.29 (d, 4a).

**[Pd($C_{35}H_{27}N_5$)]$Cl_2$** : Found % : C, 60.89; H, 3.80; N, 9.72; Cl, 9.76; Pd, 14.88, Calcd.% : C, 60.49; H, 3.92; N, 10.08; Cl, 10.20; Pd, 15.31; mol wt. Found : 680; Calcd. : 695; m.p., 158°C; $^1H$ NMR (DMSO-$d_6$) : δ 3.43 (bs,-$NCH_2$), δ 7.86-8.10 (R=Py.$C_5H_7N_3$), and aromatic protons : δ8.10 (d, 2a), δ7.86 (dd,3a), δ7.95 (d, 4a).

**[Pd($C_{36}H_{28}N_4$)]$Cl_2$** : Found.% : C, 61.92; H, 3.91; N, 7.68; Cl, 9.81; Pd, 14.95, Calcd. % : C, 62.30; H, 4.07; N, 8.07; Cl, 10.22; Pd, 15.33; mol wt. Found : 662; Calcd : 694; m.p., 141°C; $^1H$ NMR (DMSO-$d_6$) : δ3.37 (bs, -$NCH_2$), δ7.37-7.49 (R=Ph.$C_6H_8N_2$), and aromatic protons : δ7.49 (d, 2a), δ7.37 (dd, 3a),δ7.43 (d, 4a).

**Analytical Methods and Physical Measurements**

Conductivity measurements were carried out in $10^{-3}$ mol $dm^{-3}$ dimethylformamide solutions at 25°C using a Systronics type 305 conductivity bridge. Melting points were determined in capillary tubes and were uncorrected. The IR spectra of precursor and complexes were recorded, as KBr discs, using a model Nicolet Megna FTIR-550 spectrophotometer and the electronic absorption spectra of precursor and complexes is DMSO solution were recorded at 25°C using a Hitachi U-2000 spectrophotometer. Molecular weights were determined by the Rast Camphor Method[21]. The $^1H$ NMR spectra were obtained in DMSO-$d_6$ using JEOL FX 90Q spectrometer with $Me_4Si$ as an internal standard. Chemical shifts were reported in ppm. Cyclic voltamograms were recorded on EG & G park scanning potentiostate model 362. Mass spectra were recorded on a JEOL SX 102/DA-6000 mass spectrometer with fast atom bombardment and elemental analyses were performed at Central Drug Research Institute, Lucknow, India. $^{13}C$ NMR spectra were recorded in methanol, using TMS as the standard.

Palladium was determined gravimetrically[22]. Lead was estimated as lead sulphate. Nitrogen and chlorine were determined by Kjeldahl's and Volhard's methods, respectively[23].

### RESULTS AND DISCUSSION

The elemental analysis and spectral data suggested the formation of the precursor ($C_{30}H_{24}N_2O_2$) alongwith the macrocyclic complexes [Pb($C_{30}H_{24}N_2$)(R)$Cl_2$] and [Pd($C_{30}H_{24}N_2$)(R)] $Cl_2$. A proposed scheme of synthetic route for palladium complexes is given below.

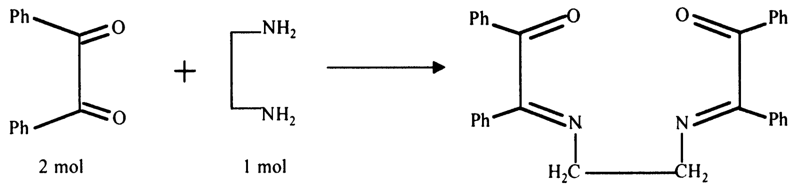

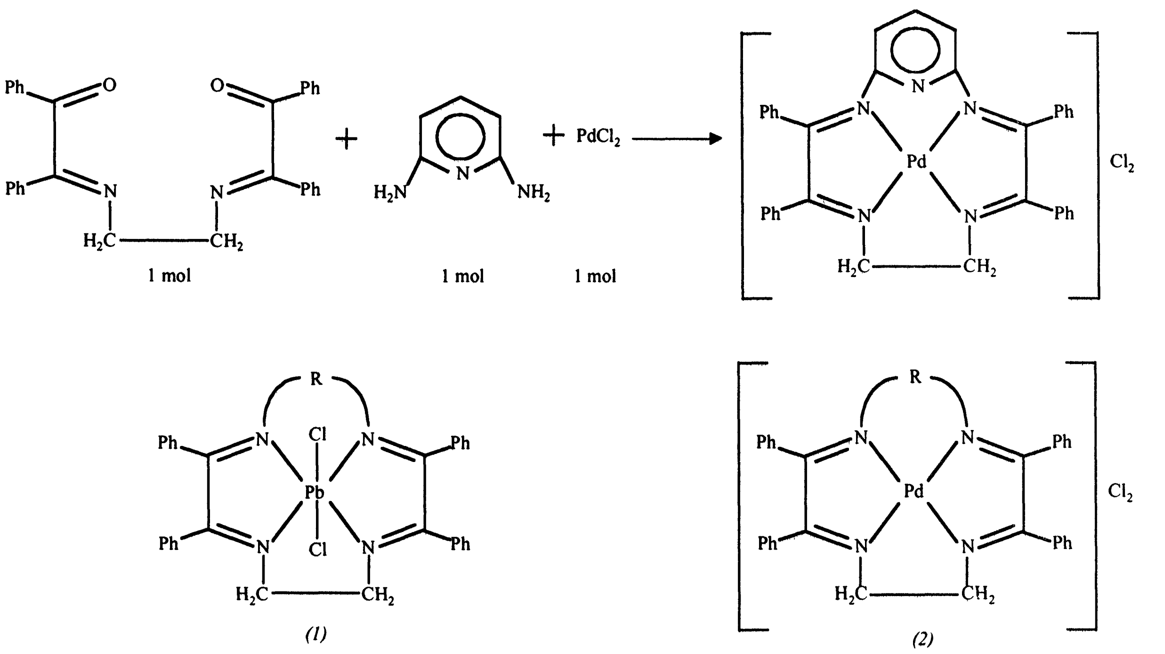

(Where R = 2,6-diaminopyridine or 1,2-phenylenediamine)

**Fig. 1**

The resulting coloured solids are stable at room temperature and non-hygroscopic. These complexes are insoluble in water but soluble in common organic solvents, DMF and DMSO without change in colour. The monomeric nature of these complexes is confirmed by molecular weight determinations. The molar conductance of the lead compounds in distilled DMF ($10^{-3}$M), are in 14-21 $cm^2$ $ohm^{-1}$ $mol^{-1}$ range, correspond to non-electrolytes while that of the palladium complexes appear in the range (206-218 $cm^2$ $ohm^{-1}$ $mol^{-1}$) expected for 1:2 electrolytes.

### SPECTRAL STUDIES

### IR Spectra

A comparative study of the IR spectra of the precursor and its complexes confirmed the formation of the macrocyclic complexes with the proposed coordination pattern. In the IR spectra of all the complexes the functional groups $NH_2$ and C=O of the starting compounds, are absent whereas characteristic bands of imine groups[24] are present. The absence of stretching and deformation vibrations of $-NH_2$ groups[25] indicates the deprotonation of macrocyclic complexes and the appearance of strong bands due to coordinated νC=N vibrations[26] in the range 1615-1598 $cm^{-1}$ are the major changes observed in the IR spectra of the macrocyclic complexes. This contention is supported by the presence of bands at 1455-1476 $cm^{-1}$ and 1360-1382 $cm^{-1}$ characteristic of benzil moiety and may be assigned to ($\nu_{asym}$ $C_6H_5$) and ($\nu_{sym}$ $C_6H_5$). The spectra do not show any change in pyridine ring vibrations and it appears that in these complexes the nitrogen atom of pyridine does not participate in coordination. Strong and sharp bands for C-H stretching and bending vibrations appear at *ca* 2820 and 1410 $cm^{-1}$, respectively[24]. The presence of an aromatic C-N band in the complexes appeared at 840 $cm^{-1}$.

The coordination of nitrogen to the metal is further supported by the appearance of new band of medium intensity in the region 445-480 $cm^{-1}$ due to νM-N vibration[27]. Further , a new band of medium intensity at 430 $\pm$ 5 $cm^{-1}$ is due to ν Pb-Cl in lead complexes.

### $^1$H NMR Spectra

The proton magnetic resonance spectra of the precursor as well as its corresponding metal complexes have been recorded in DMSO-$d_6$ using TMS as internal standard. The spectral data give some important information to conclude to the formation of macrocyclic complexes. The disappearance of the primary amino proton signal and the appearance of a singlet observed at δ3.29–3.37 ppm in the complexes may be assigned to methylene protons adjacent to nitrogen indicated that the proposed macrocyclic skeleton has been formed. The multiplate of aromatic protons were observed at δ7.24-8.10 ppm in the spectra of the macrocycle and its complexes.

**Electronic Spectra**

The electronic spectra of the metal complexes were recorded at room-temperature in distilled DMSO. The absorption maximum at 413 nm in the case of the macrocycle can be assigned to the n-π* transition of the azomethine group. The shift of this band (*ca* 12nm) in the spectra of the complexes suggest the coordination of the nitrogen to the metal ion[28]. In addition to this band, the spectra of the metal complexes exhibit bands around 281 nm and 329 nm due to π-π* electronic transitions. However, the position of these bands remains almost same as that of the macrocycle.

The electronic spectra of the palladium (II) complexes also support the said structure. Three d-d spin allowed transitions were observed, corresponding to the transition from the three lower lying 'd' levels to the empty $d_{x^2-y^2}$ orbitals. The ground state is $^1A_{1g}$ and the excited states corresponding to the above transitions are $^1A_2$, $^1B_{1g}$ and $^1E_g$ in order of increasing energy. Three d-d- bands are observed in the regions 528-536 nm, 465-480 nm and 357-370 nm in the present palladium complexes which may be assigned to $^1A_{1g} \rightarrow {}^1A_{2g}$, $^1A_{1g} \rightarrow {}^1B_{1g}$, and $^1A_{1g} \rightarrow {}^1E_g$ transitions[29,30], respectively. The electronic spectra of these complexes indicate square-planar geometry around the palladium(II) ion. These values are in close agreement with those reported earlier for the square-planar complexes[29].

**Mass Spectra**

The FAB mass spectrum of one of the metal complex was recorded on a JEOL SX 102/DA-6000 Mass Spectrometer/Data system using Argon/Xenon (6KV, 10mA) as the FAB gas. The accelerating voltage was 10 KV and the spectrum was recorded at room temperature. m-Nitrobenzyl alcohol(NBA) was used as the matrix. In the mass spectrum, the molecular ion peak of the complex $[Pb(C_{35}H_{27}N_5)Cl_2]$, appeared at *m/z* 794 $[M]^+$, 796 $[M+2]^+$ and 798 $[M+4]^+$. Some other peaks appeared at *m/z* 640, 689, 714 and 717 correspond to the $[Pb(C_{23}H_{17}N_5)Cl_2]$, $[Pb(C_{30}H_{24}N_2)Cl_2]^+$, $[Pb(C_{33}H_{23}N_3)Cl]^+$ and $[Pb(C_{30}H_{24}N_4)Cl_2]^+$species which resulted from the loss of the $C_{12}H_{10}$, $C_5H_3N_3$, $C_2H_4N_2Cl$ and $C_5H_3N$ or $C_6H_5$ fragments from the parent compound. Two peaks appeared at *m/z* 724 and 759 due to the loss of two chlorine atoms and one chlorine atom, respectively.

**Electrochemical Studies**

The solvent and base electrolyte are reported below the each cyclic voltamogram with their scan rates. All C.V. scan were recorded on EG & G parc scanning potentio state model 362. The cyclic voltametric studies showed that substituents, on the periphery of macrocyclic ring, affects the E_ and Ep values according to their nature, whether electron withdrawing or electron donating. We report here efforts to design complexes with appropriate redox behaviour of the macrocyclic complexes containing pyridine nitrogen in the macrocyclic cavity which not participating in bond formation. The redox behaviour of these macrocycles has been observed by cyclic voltametric studies. We have synthesized phenyl derivatives of $N_5$ macrocyclic complexes of Pb and Pd metal which showed the redox behaviour interestingly irreversible manner depicted in Figs. 2 and 3.

The cyclic voltametric behaviour of the macrocyclic compounds $[Pb(C_{35}H_{27}N_5)Cl_2]$ and $[Pd(C_{35}H_{27}N_5)]Cl_2$ was studied on a Pyrrolytic Graphite Electrode (PGE). These complexes showed oxidation and reduction peaks (Tables-I and II). These electrochemical properties were determined in DMSO using $LiNO_3$ (0.1M) as supporting electrolyte in conventional three electrode cell. Their electrochemical potentials were recorded v/s Saturated Calomel Electrode. The scanning procedure is illustrated in the figures. The two oxidation peaks can be assigned as ligand centre[31] and considered to be irreversible in nature and other peak if any seems on C.V. on this oxidation side may be considered prewave peak.

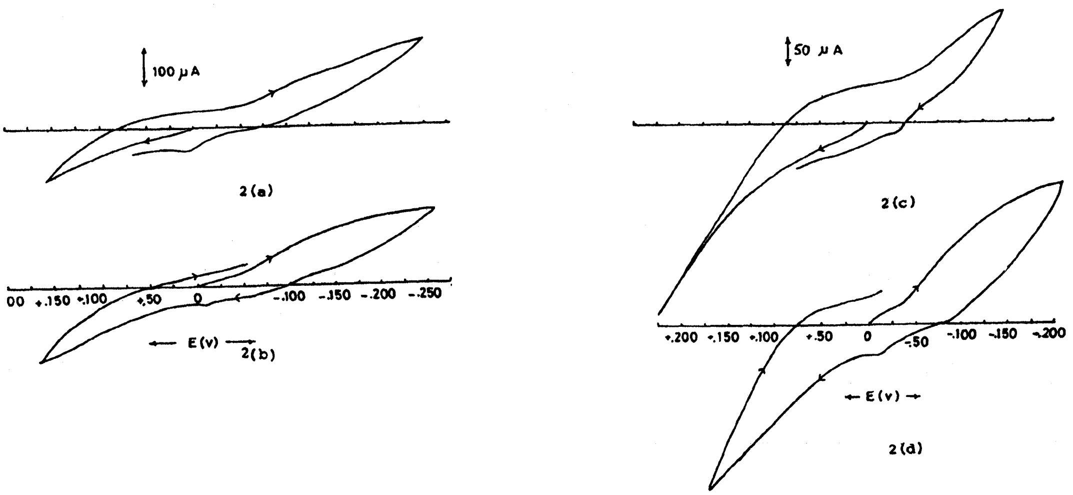

**Fig. 2**

The reduction process seems to be irreversible but the reduction products also become gradually engulfed in the prepeaks/prewave. The film growth is not observed for a large number of scans. Thus only limited studies for C.V. scan were carried out on PGE electrode. No dimerization/polymerization was observed during multiple C.V. scanning.

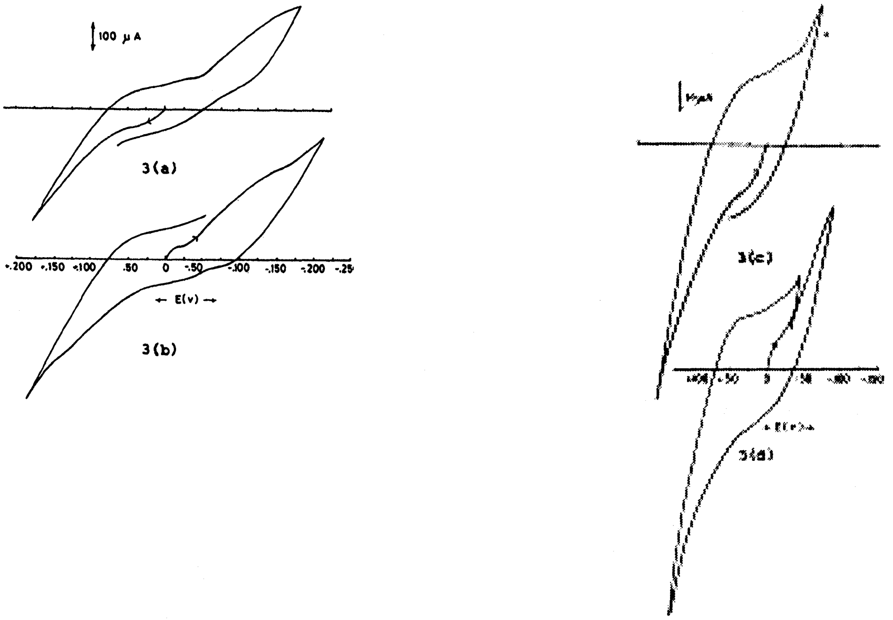

**Fig. 3**

It is noteworthy that the M-N bond distance in the $Pd^{+2}$ and $Pb^{+2}$ complexes, in contrast to cyclam is close to those for the corresponding complexes with noncyclic ligand. Thus it can be said that the present macrocyclic complex[32,33] cavity is having a little bit distortion towards "Saddle Shape" which also helps in explaining the non-formation of a M-N bond with the pyridine moiety.

**Table I : Electrochemical Data for the Lead Compound [Pb($C_{35}H_{27}N_5$)$Cl_2$]**

Concentration - 2mm in DMSO
X-axis - 250 mv
Scan Rate - 100 mv/sec.

| Compound | 1. Oxidation current – 100 μA | 2. Reduction current - 100 μA | 3. Oxidation current – 50 μA | 4. Reduction current – 50 μA |
|---|---|---|---|---|
| [Pb($C_{35}H_{27}N_5$)$Cl_2$] | One peak observed<br>Ep = + 125 mv | No peak observed | No peak observed | Two peak observed<br>$Ep_1$ = -125 mv<br>$Ep_2$ = -1000 mv. |

**Table II : Electrochemical Data for the Palladium Compound [Pd($C_{35}H_{27}N_5$)]$Cl_2$**

Concentration - 2mm in DMSO
X-axis - 250 mv
Scan Rate - 100 mv/sec.

| Compound | 1. Oxidation current – 100 μA | 2. Reduction current - 100 μA | 3. Oxidation current – 50 μA | 4. Reduction current – 50 μA |
|---|---|---|---|---|
| [Pd($C_{35}H_{27}N_5$)]$Cl_2$ | No peak observed | Two peaks observed<br>$Ep_1$ = -1375 mv<br>$Ep_2$ = -250 mv | No peak observed | One peak observed<br>$Ep_1$ = +250 mv. |

### $^{13}$C NMR Spectra

The $^{13}$ C NMR spectra of the precursor, $C_{30}H_{24}N_2O_2$ and its lead and palladium compounds have been recorded which clearly support the formation of the present compounds.

**Table III : $^{13}$C NMR Spectral Data (δ, ppm) of the Precursor and its Metal Complexes**

| Compound | Chemical Shift Values | | R | | | Aromatic Carbons | | | |
|---|---|---|---|---|---|---|---|---|---|
| | >C=N | -$CH_2$-N | >C-N | $C_b$ | $C_d$ | $C_{1a}$ | $C_{2a}$ | $C_{3a}$ | $C_{4a}$ |
| ($C_{30}H_{24}N_2O_2$) | 160.92 | 42.37 | - | - | - | 128.61 | 127.20 | 126.77 | 126.12 |
| [Pb($C_{35}H_{27}N_5$)$Cl_2$] | 154.46 | 43.92 | 141.46 | 134.18 | 137.57 | 130.60 | 129.38 | 127.09 | 126.87 |
| [Pb($C_{36}H_{28}N_4$)$Cl_2$] | 156.15 | 44.38 | 146.21 | 139.64 | 142.31 | 131.48 | 129.21 | 128.24 | 127.58 |
| [Pd($C_{35}H_{27}N_5$)]$Cl_2$ | 149.08 | 39.88 | 139.76 | 133.82 | 135.66 | 129.33 | 127.69 | 127.10 | 125.94 |
| [Pd($C_{36}H_{28}N_4$)]$Cl_2$ | 150.86 | 40.43 | 143.52 | 136.26 | 140.11 | 132.13 | 130.41 | 128.16 | 127.81 |

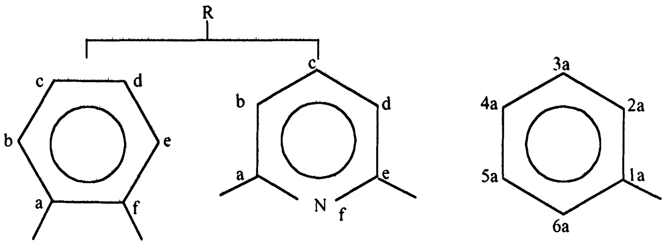

On the basis of the above evidences, structures *(1)* and *(2)* may be proposed for the lead and palladium macrocyclic complexes.

### BIOLOGICAL SCREENING

Biochemical applications have greater demand now a days. Activities of fungi and bacteria on several compounds give more important information about complexes. So it is promoted us to screen all the macrocyclic complexes and precursor to find out which part of the molecule is actually responsible for its physiological activity.

### Antifungal Activity

The antifungal activities were evaluated against *Fusarium oxysporum* and *Alternaria alternata* by the agar plate technique[34]. The compounds were dissolved in 50, 100 and 200 ppm concentrations in MeOH and then mixed with the medium. The linear growth of the fungus was obtained by measuring the diameter of the colony after 96 h. The percentage inhibition was calculated as 100(C-T)/C, where C and T are the diameters of the fungus colony in the control and test plates, respectively.

### Antibacterial Activities

Bactericidal activities were evaluated by the inhibition zone technique[35]. The nutrient agar medium (Peptones, Beef Extract, NaCl and agar-agar and 5mm diameter paper discs (Whatman No.1) were used. The compounds were dissolved in MeOH in 500 and 1000 ppm concentrations. The filter paper discs were soaked in different solution of the compounds, dried and then placed in the petriplates previously seeded with test organisms (*Escherichia coli* and *Staphylococcus aureus*). The plates were incubated for 24-30h at 29±1°C and the inhibition zone around each disc was measured. Data for fungicidal and bactericidal activities are given in Tables IV and V.

**Table IV : Fungicidal Screening Data of Macrocycle and its Complexes : Inhbition % after 96 h (conc. in ppm)**

| Compound | Inhibition after 96h (conc. in ppm) | | | | | |
|---|---|---|---|---|---|---|
| | *Fusarium oxysporum* | | | *Alternaria alternata* | | |
| | 50 | 100 | 200 | 50 | 100 | 200 |
| Standard (Bavistin) | 85 | 100 | 100 | 87 | 100 | 100 |
| ($C_{30}H_{24}N_2O_2$) | 30 | 34 | 39 | 28 | 35 | 41 |
| [$Pb(C_{35}H_{27}N_5)Cl_2$] | 33 | 42 | 50 | 31 | 45 | 52 |
| [$Pb(C_{36}H_{28}N_4)Cl_2$] | 37 | 44 | 53 | 34 | 47 | 55 |
| [$Pd(C_{35}H_{27}N_5)$]$Cl_2$ | 42 | 49 | 60 | 40 | 50 | 58 |
| [$Pd(C_{36}H_{28}N_4)$]$Cl_2$ | 39 | 47 | 57 | 37 | 48 | 54 |

**Table V: Bactricidal Screening Data of the Macrocycle and its Complexes : Inhibition (mm) after 24 h (conc. in ppm)**

| Compound | Inhibition (mm) after 24 h (conc. in ppm) | | | |
|---|---|---|---|---|
| | *Staphylococcus aureus* (+) | | *Escherichia coli* (-) | |
| | 500 | 1000 | 500 | 1000 |
| Standard (Streptomycin) | 15 | 17 | 17 | 18 |
| ($C_{30}H_{24}N_2O_2$) | 5 | 8 | 4 | 7 |
| [$Pb(C_{35}H_{27}N_5)Cl_2$] | 6 | 9 | 8 | 10 |
| [$Pb(C_{36}H_{28}N_4)Cl_2$] | 8 | 9 | 6 | 9 |
| [$Pd(C_{35}H_{27}N_5)$]$Cl_2$ | 12 | 12 | 10 | 11 |
| [$Pd(C_{36}H_{28}N_4)$]$Cl_2$ | 10 | 11 | 9 | 12 |

### RESULTS

The macrocycle and its complexes were screened for their antifungal activity against *Fusarium oxysporum* and *Alternaria alternata*. Their antibacterial properties were also evaluated by testing them against *Escherichia coli* and *Staphylococcus aureus*. The experimental results show that there is an increase in the toxicity of the complexes as compared to the precursor. The results recorded from the biological activity were also further compared with the standard fungicide *Bavistin* and conventional bactericide *Streptomycin*. It was important to note that the complexes show more inhibitory effects and inhibit the growth of fungi and bacteria to a greater extent than the macrocycle. Solubility and concentration of the compounds play vital roles in ascertaining the extent of inhibition. This can be well ascribed to Tweedy's chelation theory[36]. According to Lawrence et.al[37] the biocidal properties of complexes against various microorganisms depend on the impermeability of the cell. The bactericidal activity of complexes was greater towards gram positive strain as compared to gram negative strain.

### ACKNOWLEDGEMENT

The authors are thankful to the Council of Scientific and Industrial Research and the Government of India, Ministry of Science and Technology, New Delhi, India for the financial assistance.

### REFERENCES

1. M.W. Hosseini, J.M. Lehn, S.R. Duff, K. Gu and M.P. Mertes, *J. Org. Chem.*, **52**, 1662 (1987).
2. R.M. Izan, J.S. Brandshaw, S.A. Nielsen, J.D. Lamb, J.J. Christensen and D. Sen, *Chem. Rev.*, **85**, 271 (1985).
3. C.J. Jones, *Chem. Soc. Rev*, **27**, 289 (1998).
4. D. Philp, J.F. Stoddart, *Angrew, Chem.*, **108**, 1242 (1996); *Angew. Chem. Int. Ed. Engl.*, **35**, 1154 (1996).
5. A.W. Maverick, M.L. Ivie, J.H. Waggenspack, F.R. Fronczek, *Inorg. Chem.*, **29**, 2403 (1990).
6. H. Sleiman, P. Baxter, J.-M. Lehn, K. Rissanen, *J. Chem. Soc., Chem. Commun.*, 715 (1995).
7. P. Baxter, J.-M. Lehn, J. Fischer, M.-T. Youinou, *Angew. Chem.*, **106**, 2432 (1994); *Angew. Chem. Int. Ed. Engl.* **33**, 2284(1994).
8. P.Baxter, J.-M. Lehn, A. Decian, J. Fischer, *Angew. Chem.* **105**, 92 (1993).
9. J.Manna,J. A. Whiteford. P.J.Stang *J.Am. Chem. Soc.*, **118**, 8731 (1996).
10. P.J. Stang, *Chem. Eur. J.*, **4**, 19(1998)
11. M.Albrecht, *Chem. Soc .Rev.*, **27**,281 (1998).
12. A. McAuley and C.Xu, *Inorg. Chem.*, **31**,5549 (1992).
13. M.P. Suh, G.-Y. Kong and I.-S.Kim, *Bull. Korean Chem. Soc.*, **14**,439 (1993).

14. I.M. Alkinson, P.J. Baillie, N.Choi, L. Fabbrizzi, L.F. Lindoy, M.McPartlin and P.A. Tasker, *J. Chem. Soc., Dalton Trans.*, 3045 (1996).
15. Zhao Zhong Jiang and A.Sen, *J.Am. Chem. Soc.,* **117**,4455 (1995).
16. Sr. Cherayath, J.Alice and C.P. Prabhakaran, *Transition Met. Chem.*, **15**,449 (1990).
17. H. Urata, M.Tanaka and T. Fuchikami, *Chem. Lett.,* **4**,751 (1987).
18. M. Momentean and C.A. Reed, *Chem. Rev.,* **94**, 585 (1994).
19. B. Singh, R.N. Singh and R.C. Aggarwal, *Polyhedron*, **4**, 40 (1985).
20. K. Dey, D. Bandyopadhyay, K.K. Nandi, S.N. Poddar, G. Mukhopadhyay and G.B. Kauffman, *Synth. React. Inorg. Met.-Org. Chem.*, **22**, 1111 (1992).
21. A.I.Vogel, "*A Text-Book of Practical Organic Chemistry*", 4th Edition, Longmans, ELBS London, p. 232 (1978).
22. A.I. Vogel, "*Text-Book of Quantitative Inorganic Analysis*", Longmans Green, ELBS, London, p.512 (1962).
23. A.I. Vogel, "*A Text-Book of Inorganic Analysis*", Longmans, Green and CO., London, (1968).
24. N.B. Colthup, L.H. Dally and S.E. Wiberley, "*Introduction to Infrared and Raman Spectroscopy*" (Academic Press, New York), (1964).
25. M. Shakir, S.P. Varkey and O.S.M. Nasman, *Indian J. Chem.*, **35A**, 671 (1996).
26. B.K. Coltrain and S.C. Jackhls, *Inorg. Chem.*, **20**, 2032 (1991).
27. A. Kumari, J.P. Tandon and R.V. Singh, *Appl. Organomet. Chem.,* **7**, 655 (1993).
28. K. Singh, P. Dixit, R.V. Singh and J.P. Tandon, *Main Group Met. Chem.,* **12**, 155 (1989).
29. H.B. Gray and C.J. Ballhousen, *J. Am. Chem. Soc.,* **85**, 260 (1963).
30. R.V. Singh, K. Sharma and N. Fahmi, *Transition Met. Chem.,* **24**, 562 (1999).
31. C.L. Bailey, R.D. Bereman, D.P. Rilema, R. Nowak, *Inorg. Chem.*, **23**, 3956 (1984).
32. Waknine, M.J. Merg., J.F. Endicoatt, L.A. Ochrymowycz, *Inorg. Chem.,* **30**, 3691 (1991).
33. V.J. Thom, C.T. Fox, J.C.A. Baryens, R.D. Hancock, *J. Am. Chem. Soc.*, **106**, 5947 (1984).
34. J.G. Horsfall, *Bot. Rev.*, **419** (1945).
35. H.H. Thornberry, *Phytopathology*, **40**, 419 (1950).
36. B.G. Tweedy, *Phytopathology*, **55**, 910 (1964).
37. P.G. Lawrence, P.L. Harold and O.G. Francis, *Antibiotic and Chemotherapy*, **5**, 1597 (1980).

**Received: July 5, 2000 - Accepted: September 7, 2000 -**
**Received in revised camera-ready format: October 11, 2000**

